# Body Under Attack: Disseminated Varicella-Zoster Virus Infection

**DOI:** 10.7759/cureus.103696

**Published:** 2026-02-16

**Authors:** Ana M Oliveira, Joana Luís, Marco Fernandes, Rafael Curto, João Gonçalves Pereira

**Affiliations:** 1 Intensive Care Unit, Unidade Local de Saúde (ULS) do Estuário do Tejo, Vila Franca de Xira, PRT

**Keywords:** diagnosis, disseminated infection, high index suspicion, immunocompromised, varicella-zoster virus

## Abstract

Varicella-zoster virus (VZV) infection is typically benign and self-limiting. In adults, it most commonly manifests as a localized cutaneous infection. However, in immunocompromised individuals, VZV can undergo hematogenous dissemination and cause severe visceral involvement, including pulmonary, hepatic, pancreatic, cardiac, and central nervous system complications. Disseminated disease may be further complicated by multiorgan failure, acute respiratory distress syndrome (ARDS), and coagulopathy and is associated with significant mortality.

We report a 64-year-old man who presented to the emergency department with fever and abdominal pain, followed by a generalized rash with cephalocaudal progression involving the trunk and upper limbs. He was diagnosed with disseminated VZV infection, which was complicated by hepatitis, acute pancreatitis, and severe pneumonia, progressing to ARDS. The patient was admitted to the intensive care unit (ICU) and started on invasive mechanical ventilation. Early initiation (2nd day after ICU admission) of intravenous acyclovir was critical to a favorable clinical outcome, allowing discharge from the ICU on the 10th day.

Disseminated VZV infection may initially present with visceral manifestations, particularly abdominal pain, which can precede or occur in the absence of a typical vesicular skin rash, leading to diagnostic delay. Although VZV pneumonia complicated by ARDS is rare, it carries high morbidity and mortality. Early recognition, prompt antiviral therapy, and supportive intensive care management are crucial to improve outcomes. This case highlights the importance of maintaining a high index of suspicion for VZV infection in critically ill patients with unexplained systemic or respiratory severe deterioration, particularly in those with underlying immune dysfunction. Prompt recognition and treatment, as well as biopsy of the lesions, are essential to improve the prognosis of these immunocompromised patients at high risk for atypical infectious diseases.

## Introduction

Varicella-zoster virus (VZV) infection is typically a benign and self-limited disease in immunocompetent hosts. However, in immunocompromised patients, it may follow an aggressive and potentially fatal course, particularly when visceral dissemination occurs [1,2]. Severe manifestations are well described in patients with hematologic malignancies, solid organ or stem cell transplantation, human immunodeficiency virus (HIV) infection, and those receiving immunosuppressive therapies, including corticosteroids [1-3].

VZV is a neurotropic alpha-herpesvirus that establishes lifelong latency in sensory and autonomic ganglia after primary infection. Reactivation or uncontrolled viral replication occurs mainly in the context of impaired cell-mediated immunity, which plays a central role in viral containment [2,4]. In critically ill patients, even in the absence of classical immunosuppressive conditions, immune dysregulation and critical-illness-associated immunoparalysis may predispose to viral reactivation or dissemination, thereby contributing to delayed recognition and poorer outcomes [5,6].

Disseminated VZV infection is defined by widespread cutaneous lesions and/or visceral organ involvement, including the lungs, liver, pancreas, gastrointestinal tract, and central nervous system. Among these, pulmonary involvement is the most frequent and severe in adults, often progressing rapidly to acute respiratory distress syndrome (ARDS) and multiorgan failure [7,8]. Reported mortality rates for severe VZV pneumonia in adults admitted to the intensive care unit (ICU) range from 20% to over 40%, particularly in patients requiring invasive mechanical ventilation (IMV) [7].

A major diagnostic challenge lies in the frequent absence of early or typical cutaneous manifestations. In immunocompromised or critically ill patients, visceral involvement may precede skin lesions by several days or occur in their complete absence, a phenomenon described as “zoster sine herpete” [2,4,9]. Consequently, initial clinical presentations may mimic more common ICU diagnoses, such as bacterial sepsis, acute pancreatitis, or noninfectious causes of respiratory failure, leading to diagnostic delay and delayed antiviral therapy [5,8,9].

Laboratory diagnosis is also challenging. Serological testing is often unhelpful in the acute phase, particularly during primary infection or early reactivation, as IgM responses may be absent and IgG seroconversion may be delayed [1,2,5]. Polymerase chain reaction (PCR) testing of blood, respiratory samples, or tissue biopsies has therefore become the diagnostic gold standard in suspected disseminated disease, especially in critically ill patients with atypical presentations [2,4].

Early initiation of intravenous acyclovir remains the cornerstone of treatment in severe VZV infection and has significantly reduced mortality compared to the pre-antiviral era [2,3]. Nonetheless, despite appropriate antiviral therapy, outcomes remain poor in patients with advanced organ dysfunction, severe hypoxemia, or profound immunosuppression [6,7]. The role of adjunctive therapies, such as corticosteroids, in VZV pneumonia remains controversial, with conflicting evidence regarding their impact on mortality and secondary infections [5,7].

We report a case of disseminated VZV infection in a critically ill adult patient admitted to the ICU, characterized by atypical initial presentation, rapid progression to multiorgan failure, and delayed cutaneous manifestations. This case highlights the diagnostic challenges posed by VZV infection in the ICU setting. It underscores the need for a high index of suspicion, early molecular diagnostics, and prompt antiviral therapy in critically ill patients with unexplained systemic or respiratory deterioration.

## Case presentation

A 64-year-old man presented to the emergency department (ED) with a three-day history of acute abdominal pain. He was discharged with symptomatic treatment, which provided partial relief. Twenty-four hours later, he returned to the ED due to the onset of a generalized rash, initially involving the face and subsequently progressing in a cephalocaudal distribution to the trunk, upper limbs, and thighs. The rash was non-pruritic. He also reported bilateral periorbital edema, asthenia, and anorexia, denying other symptoms.

His past medical history was remarkable for Hodgkin’s lymphoma under active chemotherapy, resulting in secondary immunodeficiency, and known bronchiectasis chronically colonized with multidrug-resistant *Klebsiella pneumoniae*.

On physical examination, the patient appeared drowsy and ill. He had exuberant bilateral periorbital edema with conjunctival hyperemia. Respiratory examination revealed dyspnea, tachypnea (respiratory rate 22 cycles per minute), and hypoxemia, with peripheral oxygen saturation of 90% on room air. Hemodynamically, he was hypotensive (blood pressure 89/49 mmHg) and febrile (temperature 38.7°C). Dermatological examination revealed extensive, bilateral, and symmetrical vesiculobullous lesions at different stages of evolution, including necrotic, umbilicated, and coalescing, non-pruritic lesions with hemorrhagic features, involving the oral mucosa, face, trunk, upper limbs, and thighs. Additionally, whitish, non-detachable plaques were observed on the tongue and oropharynx. No other relevant findings were noted.

Initial laboratory evaluation (Table 1) revealed anemia (hemoglobin 11.6 g/dL), severe lymphopenia (100 cells/mm³), and thrombocytopenia (97,000/mm³). Renal function showed mild impairment (serum creatinine 1.06 mg/dL, urea 52 mg/dL). Liver function tests demonstrated a mixed cytolytic and cholestatic pattern (AST 262 U/L, ALT 238 U/L, GGT 551 U/L, alkaline phosphatase 259 U/L, total bilirubin 0.5 mg/dL, LDH 435 U/L). CRP was elevated, 9.06 mg/dL. Serum lipase was increased (586 U/L). A contrast-enhanced thoraco-abdominal CT scan revealed bronchiectasis in the lower pulmonary lobes, multiple bilateral pulmonary consolidations, and diffuse edematous acute pancreatitis.

**Table 1:** Complementary diagnostic tests performed in the ED Ig: immunoglobulin, HIV: human immunodeficiency virus, TPHA: Treponema pallidum hemagglutination assay, FTA-ABs: fluorescent treponemal antibody absorption, PCR: polymerase chain reaction, DNA: deoxyribonucleic acid, SARS-CoV-2: severe acute respiratory syndrome coronavirus 2, ED: emergency department

Hemoglobin	11.6	g/dL	13.0-17.0
Hematocrit	33.4	%	40.0-50.0
Mean corpuscular volume (MCV)	84.8	fL	80.0-97.0
Mean corpuscular hemoglobin (MCH)	29.4	pg	27.0-32.0
Mean corpuscular hemoglobin concentration (MCHC)	34.7	g/dL	32.0-36.0
Red cell distribution width (RDW)	17.7	%	11.6-14.0
White blood cells	5.2	×10³/µL	4.0-10.0
Neutrophils	4.91 (94.4%)	×10³/µL	1.5-8.0
Lymphocytes	0.10 (1.9%)	×10³/µL	0.8-4.0
Monocytes	0.11 (2.1%)	×10³/µL	0.0-1.2
Eosinophils	0.01 (0.2%)	×10³/µL	0.0-0.3
Basophils	0.02 (0.4%)	×10³/µL	0.0-0.3
Platelets	97	×10³/µL	150-400
Coagulation			
Prothrombin time (PT)	16.9	s	11-13
International normalized ratio (INR)	1.43	-	<1.2
Activated partial thromboplastin time (aPTT)	32	s	22.1-28.1
Fibrinogen	332	mg/dL	170-420
Renal function and electrolytes			
Urea	52	mg/dL	<50
Creatinine	1.06	mg/dL	0.70-1.30
Sodium	128	mmol/L	136-145
Potassium	3.77	mmol/L	3.5-5.1
Chloride	94	mmol/L	98-107
Liver function and enzymes			
Aspartate aminotransferase (AST)	262	U/L	15-37
Alanine aminotransferase (ALT)	238	U/L	16-63
Gamma-glutamyl transferase (GGT)	551	U/L	15-85
Alkaline phosphatase (ALP)	259	U/L	50-136
Total bilirubin	0.5	mg/dL	<1.0
Lactate dehydrogenase (LDH)	435	U/L	85-227
Creatine kinase (CK)	70	U/L	39-308
Inflammatory and protein markers			
C-reactive protein (CRP)	9.06	mg/dL	0.06-1.00
Pancreatic stone protein	268	ng/mL	<300
Albumin	2.08	g/dL	3.4-5.0
Pancreatic and metabolic parameters			
Amylase	89	U/L	25-115
Lipase	586	U/L	73-393
Calcium	6.7	mg/dL	8.5-10.1
Phosphate	3.6	mg/dL	2.5-4.9
Magnesium	1.8	mg/dL	1.8-2.4
Cardiac and other markers			
High-sensitivity troponin I	13.6	pg/mL	<72
Serum tryptase	5.6	µg/L	<11.4
Serological tests			
Ab. anti-herpes simplex 1 (VHS)	IgG positive; IgM negative		
Ab. anti-herpes simplex 2 (VHS)	IgG and IgM negative		
Ab. anti-herpes 6	IgG positive; IgM negative		
Ab. anti-cytomegalovirus (CMV)	IgG positive; IgM negative		
Ab. anti-Epstein-Barr virus (EBV)	IgG positive		
Ab. anti-varicella-zoster (VZV)	IgG and IgM negative		
Enterovirus	Negative		
Ab. anti-*Coxiella burnetii*	IgG positive		
Ab. anti-*Rickettsia conorii*	IgG positive; IgM negative		
Ab. anti-*Bartonella*	IgG negative		
Ab. anti-*Brucella*	IgG negative		
Ab. anti-*Leishmania*	IgG, IgM, and IgA negative		
Ab. anti-*Rubéola*	IgG and IgM negative		
Ab. anti-*Leptospira interrogans*	IgG and IgM negative		
Treponema pallidum	IgM, FTA-ABs, TPHA negative		
Ab. anti-HIV 1 and 2	Negative		
Ab. anti-*Toxoplasma gondii*	IgG and IgM negative		
Other tests			
Respiratory virus panel (PCR) – influenza A/B; parainfluenza 1/2/3/4; rhinovirus; adenovirus; coronavirus; bocavirus; respiratory syncytial virus A/B; metapneumovirus; *Bordetella pertussis/parapertussis*; *Mycoplasma pneumoniae*	Negative		
Herpes virus (DNA) panel (exudate vesicles)	VZV positive; CMV, HSV 1 and 2, EBV, *Herpesvirus hominis* 6 and 7, and enterovirus negative		
Pharyngeal *Streptococcus A*	Negative		
PCR SARS-CoV-2	Negative		
Urinary antigen tests - *Streptococcus pneumoniae*	Positive		
Urinary antigen tests - *Legionella pneumophila*	Negative		

**Figure 1:**
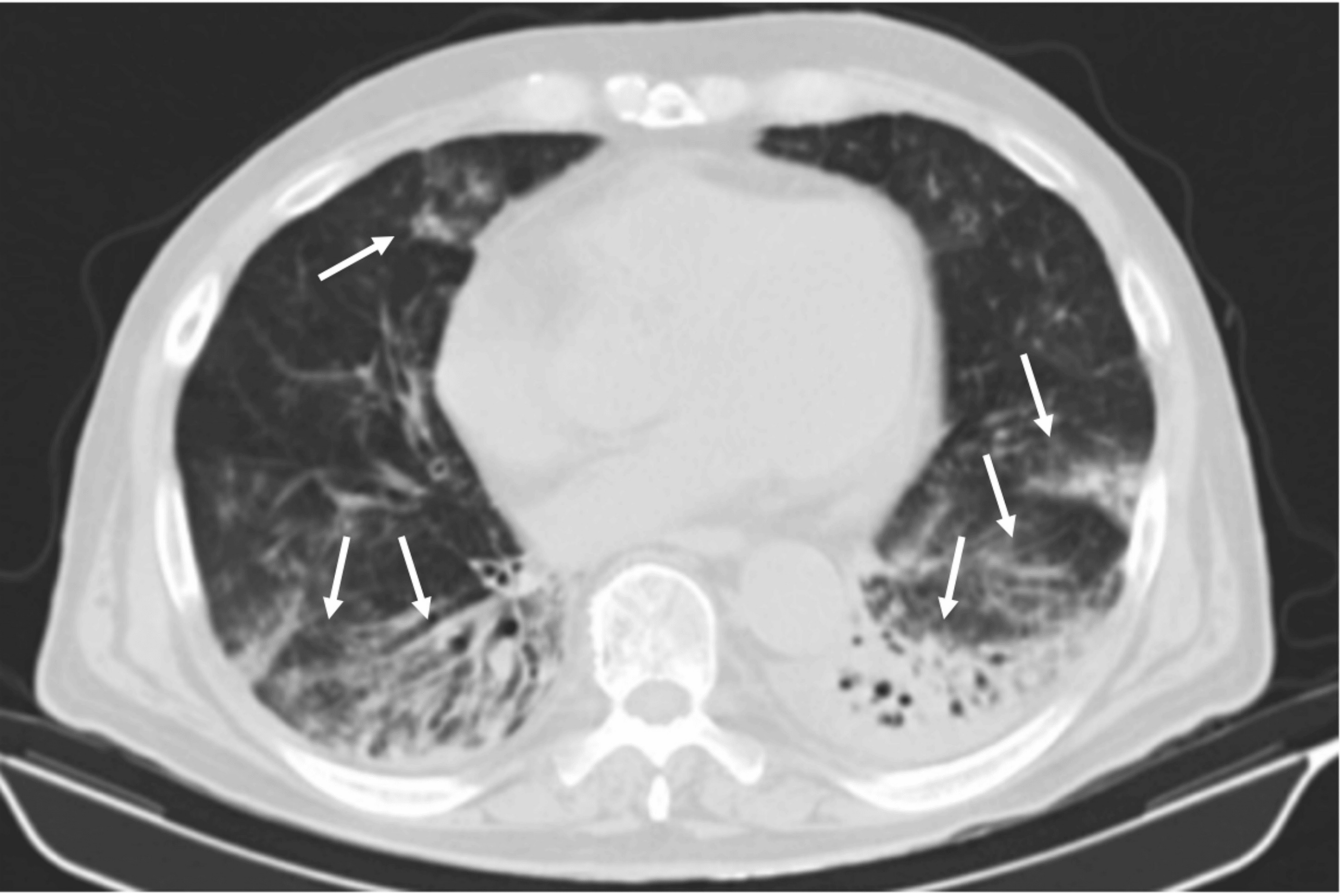
Chest CT scan with bilateral pneumonia of VZV infection showing multiple bilateral pulmonary consolidations (arrows) CT: computed tomography, VZV: varicella-zoster virus

**Figure 2:**
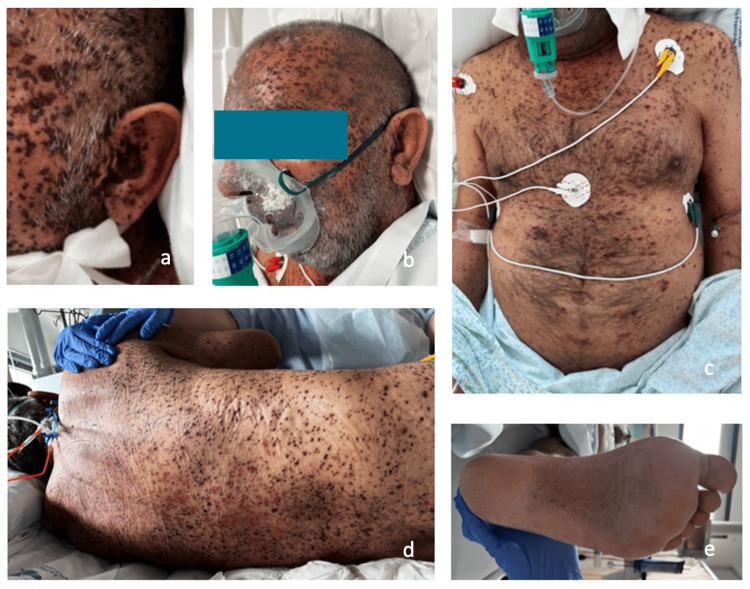
(a-e) Skin lesions of disseminated VZV infection showing multiple vesicular-bullous lesions in different stages of development, necrotic, umbilicated, and coalescent. VZV: varicella-zoster virus

Persistent hypotension required vasopressor support with norepinephrine (maximum dose 15 µg/min). He was admitted to the ICU. On the second ICU day, worsening respiratory symptoms were noted, including persistent tachypnea and productive cough. He remained febrile. Laboratory reassessment showed rising CRP (13.68 mg/dL), with a normal pancreatic stone protein level (268 ng/mL; normal <300 ng/mL). Urinary antigen testing was positive for Streptococcus pneumoniae. A chest CT scan suggested bilateral pneumonia (Figure 1), and meropenem (1g tid) was started. No clinical improvement was noticed, and the patient progressed to septic shock. At this stage, extensive and rapidly progressive hemorrhagic skin lesions affected the entire body (Figure 2a-2e).

Histopathological examination of a skin biopsy demonstrated dermatitis. Immunohistochemistry was positive for herpesvirus. VZV DNA was identified in vesicles and blood, while both VZV IgM and IgG were negative at admission (48 hours after the first appearance of skin lesions), along with repeated PCR testing in blood.

Considering the clinical presentation, comorbidities, distribution and morphology of the skin lesions, and visceral involvement, a diagnosis of disseminated primary VZV infection complicated by hepatitis, acute pancreatitis, and pneumonia was established. The patient was started on intravenous acyclovir (10 mg/kg tid) for 10 days and intravenous immunoglobulin, 1 g/kg/day for two days. Antifungal therapy with intravenous fluconazole (150 mg/day) was added, along with topical fusidic acid and zinc oxide for skin care.

On the fourth ICU day, progressive hypoxemic respiratory failure was noted, with increasing oxygen requirements. Global respiratory failure developed and required endotracheal intubation and IMV; the clinical and radiological pattern was suggestive of ARDS. Adjunctive systemic corticosteroid therapy with intravenous methylprednisolone (1 mg/kg/day) was initiated for 10 days.

**Figure 3:**
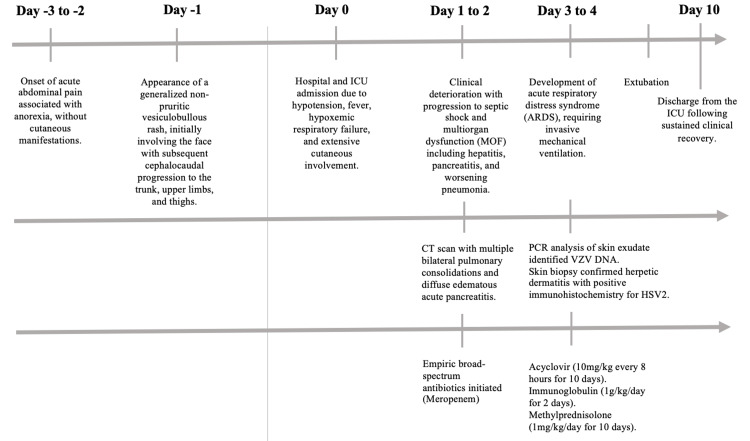
Timeline summarizing symptom onset, rash appearance, organ involvement, diagnostic milestones, therapeutic interventions, and clinical outcomes in a patient with disseminated VZV infection complicated by acute pancreatitis, hepatitis, pneumonia, and ARDS ICU: intensive care unit, ARDS: acute respiratory distress syndrome, MOF: multiorgan failure, CT: computed tomography, PCR: polymerase chain reaction, VZV: varicella-zoster virus, DNA: deoxyribonucleic acid, HSV2: herpes simplex virus type 2

*Staphylococcus hominis* bacteremia associated with an intravenous line was treated with intravenous vancomycin for five days. Transthoracic echocardiography excluded infective endocarditis. Clinical evolution was favorable thereafter. The patient was successfully extubated after three days of IMV and discharged from the ICU on the 10th day (Figure 3).

## Discussion

We present a case of severe disseminated VZV infection with visceral involvement in an immunosuppressed patient. The initial absence of cutaneous vesicular lesions delayed the diagnosis. Prompt initiation of antiviral therapy enabled full recovery.

This case illustrates key aspects of disseminated VZV infection. In adults, disseminated VZV infection often follows a biphasic course, with initial nonspecific systemic symptoms such as fever, malaise, and abdominal pain, followed by rapid progression to visceral organ involvement and, only later, cutaneous dissemination. Abdominal pain, particularly in immunocompromised patients, is commonly an early manifestation of VZV infection and may reflect pancreatic, hepatic, or visceral nervous system involvement, even before the appearance of vesicular skin lesions [2,4,9]. This temporal dissociation between visceral symptoms and rash represents an important early warning sign that should prompt consideration of VZV in high-risk patients.

This pattern was noted in our patient; clinical manifestations suggesting visceral involvement but without a typical vesicular rash contributed to a diagnostic delay and even to hospital discharge, a scenario repeatedly described [4,9].

Pulmonary involvement represents the most severe and prognostically relevant manifestation of disseminated VZV in adults. It frequently progresses to ARDS, IMV, and substantial mortality [7]. Disease severity and mortality appear to be driven more by hypoxemia, organ failure, and the delay in starting antiviral therapy than by the underlying cause of immunosuppression itself [7,8].

This case also highlights an important diagnostic pitfall. In our patient, a positive pneumococcal urinary antigen test, likely unrelated to the clinical scenario, was misleading and delayed the diagnosis of VZV pneumonia. The positive pneumococcal urinary antigen test initially suggested a diagnosis of bacterial pneumonia, illustrating how concurrent or incidental microbiological findings may mislead clinical reasoning in critically ill patients [5,8]. In fact, the risk of a false-positive pneumococcal urinary antigen test is well known [10], and clinical interpretation of microbiological results should always be supported by strong clinical reasoning. The rapid progression of multiorgan dysfunction, severe lymphopenia, and atypical hemorrhagic skin lesions should have reinforced suspicion for viral dissemination. Explicit recognition of such misleading findings is crucial to improving diagnostic vigilance in similar cases.

Diagnostic limitations play a central role in delayed recognition. Initial serological testing is often negative, reflecting the limited sensitivity of IgM and IgG assays in early or disseminated disease [1,2,5], likely due to impaired cellular immunity. PCR-based detection of VZV in blood, respiratory samples, or tissue remains the most reliable diagnostic modality and should be pursued early when VZV is suspected [2,4], as in our patient. Early multidisciplinary discussion may facilitate timely diagnosis. In our patient, varicella infection was confirmed by detection of VZV DNA in vesicles and blood.

In contrast, both VZV IgM and IgG were negative at admission (48 hours after the first appearance of skin lesions), reflecting impaired humoral response in the context of lymphoma and chemotherapy-induced immunodeficiency [1-3]. The vesicle content tested positive for VZV DNA, whereas subsequent PCR testing of blood was negative, underscoring that molecular confirmation from lesion samples is particularly valuable in immunocompromised hosts [2,4]. These findings illustrate the limited reliability of serology in this population and reinforce PCR as the diagnostic modality of choice in suspected disseminated disease.

The role of corticosteroids in severe VZV pneumonia and ARDS remains controversial. While some case reports and small series suggest potential benefit in reducing inflammatory lung injury, larger ICU cohorts have not demonstrated a clear mortality benefit and have reported higher rates of secondary infections and prolonged mechanical ventilation [5,7]. In our case, pulmonary involvement progression, despite adequate antiviral therapy, led to our use of high-dose corticosteroids to account for a possible pulmonary excess inflammatory response [11]. Rapid reversal of ARDS was noted.

From a therapeutic perspective, the sequence and timing of interventions were critical. Empirical broad-spectrum antibiotics were initiated at ICU admission due to suspected bacterial coinfection, followed by targeted antiviral therapy once VZV was suspected, together with intravenous immunoglobulin and supportive organ replacement strategies. Mechanical ventilation was required on the fourth ICU day due to progression to ARDS. This structured escalation underscores the importance of early antiviral therapy, combined with timely supportive intensive care management, in achieving favorable outcomes [2,7].

Consequently, VZV should be considered in the differential diagnosis of unexplained sepsis, acute respiratory failure, or multiorgan dysfunction in critically ill high-risk patients, even in the absence of the typical cutaneous vesicular lesions [5,8,9].

Mortality from disseminated VZV remains significant, particularly in patients with advanced organ failure or profound immune dysfunction [6,7]. Vaccination with live-attenuated VZV vaccines is contraindicated in many immunocompromised patients, and evidence for antiviral prophylaxis in ICU populations is limited [2,3]. Nonetheless, increased awareness of viral infections in the ICU, alongside strict infection control measures and early diagnostic testing, may help mitigate morbidity and mortality.

Disseminated VZV in adults, particularly in those with hematologic malignancies or chemotherapy-related immunosuppression, may present initially with abdominal pain and systemic deterioration, rather than classic dermatological findings, demanding a high index of suspicion and early molecular investigation to avoid potentially fatal delays [2,6,9].

## Conclusions

Disseminated VZV infection is a rare but life-threatening condition. Atypical presentations without skin involvement, limited utility of blood tests, and rapid progression to respiratory failure are common. Molecular confirmation of vesicular content via PCR testing is crucial to avoid fatal delays. This case highlights the need for heightened clinical suspicion, careful interpretation of potentially misleading findings, early use of molecular diagnostics and biopsy, and prompt initiation of antiviral therapy combined with appropriate intensive care support.

## References

[1] Wiegering V, Schick J, Beer M (2011). Varicella-zoster virus infections in immunocompromised patients - a single centre 6-years analysis. BMC Pediatr.

[2] Gershon AA, Breuer J, Cohen JI (2015). Varicella zoster virus infection. Nat Rev Dis Primers.

[3] Masaoka T, Hiraoka A, Teshima H, Tominaga N (1993). Varicella-zoster virus infection in immunocompromised patients. J Med Virol.

[4] Mueller NH, Gilden DH, Cohrs RJ, Mahalingam R, Nagel MA (2008). Varicella zoster virus infection: clinical features, molecular pathogenesis of disease, and latency. Neurol Clin.

[5] Hagiya H, Kimura M, Miyamoto T, Otsuka F (2013). Systemic varicella-zoster virus infection in two critically ill patients in an intensive care unit. Virol J.

[6] Malherbe J, Iachkine J, du Cheyron D, Valette X (2020). Diffuse varicella zoster virus reactivation in critically ill immunocompromised patient. Intensive Care Med.

[7] Mirouse A, Vignon P, Piron P (2017). Severe varicella-zoster virus pneumonia: a multicenter cohort study. Crit Care.

[8] Ueno H, Hayashi M, Nagumo S (2021). Disseminated varicella-zoster virus infection causing fatal pneumonia in an immunocompromised patient with chronic interstitial pneumonia. Intern Med.

[9] Picod A, Corre E, Maury E, Duriez P, Hoyeau N, Coppo P (2017). Acute pancreatitis in immunocompromised patients: beware of varicella zoster virus primo-infection. Clin Case Rep.

[10] Blaschke AJ (2011). Interpreting assays for the detection of Streptococcus pneumoniae. Clin Infect Dis.

[11] Aguilera ER, Lenz LL (2020). Inflammation as a modulator of host susceptibility to pulmonary influenza, pneumococcal, and co-infections. Front Immunol.

